# History of Circumcision

**Published:** 1892-04

**Authors:** 


HISTORY OF CIRCUMCISION FROM THE EARLIEST TIMES TO THE PRESENT. By P. C. Remondino, M. D. Philadelphia (1231 Filbert St.), and London. F. A. Davis, Publisher, 1891. Price, cloth, $1,25; paper, 50 cents, net.

This most interesting and clever book constitutes No. 11 of "The Physicians' and Students' Ready Reference Series," most of the numbers of which have been from time to time noticed in this journal. The present volume gives the moral and physical reasons for the performance of circumcision, and incidentally gives the history of eunuchism, hermaphrodism, etc., and of the different operations practiced upon the prepuce. Its main object is to advocate the universal practice of circumcision as a prophylactic and a hygienic measure.

The writer says, in his preface: "The operation of circumcision, with its pains, annoyances, possible and probable dangers, sinks into the most trifling insignificance, in comparison to some of the results that are daily observed as the tribute that is paid by the unlucky and unhappy wearer of a prepuce for the privilege of possessing such an appendage. * * * The practice is now much more prevalent than is supposed, as there are many Christian families where the males are regularly circumcised soon after birth, who simply do so as a hygienic measure. For the benefit of these, who may congratulate themselves upon the dangers and annoyances that they and their families have escaped, and for the benefit of those who would run into these dangers but for timely warning, this book has been especially written."

At page 290 he says: "It would seem really as if a prepuce were a dangerous appendage at any time, and life-insurance companies should class the wearer of a prepuce under the head of hazardous risks; for a circumcised laborer in a powder-mill, or a circumcised brakeman or locomotive engineer, runs actually less risk than an uncircumcised tailor or watchmaker. They recognize the danger that lurks in a stricture, but what a prepuce can and does do they entirely ignore."

These quotations will serve to give an idea of what a high value is put upon circumcision by the author, and how he should have expanded his ideas to the dimensions of a book of 350 pages. The history of circumcision given is exhaustive, and is very interesting, especially in view of the obscurity of the subject. This interest is heightened, and the book rendered piquant by the clever style of the author, his clearness, accuracy, sly humor, and covert wit. The different methods for the performance of the operation are very elaborate, but they could have been made clearer by the addition of diagrams. Altogether it is an admirable treatise. W. M. J.

